# The Structural Basis for a Transition State That Regulates Pore Formation in a Bacterial Toxin

**DOI:** 10.1128/mBio.00538-19

**Published:** 2019-04-23

**Authors:** Kristin R. Wade, Sara L. Lawrence, Allison J. Farrand, Eileen M. Hotze, Michael J. Kuiper, Michael A. Gorman, Michelle P. Christie, Santosh Panjikar, Craig J. Morton, Michael W. Parker, Rodney K. Tweten

**Affiliations:** a Department of Microbiology and Immunology, University of Oklahoma Health Sciences Center, Oklahoma City, Oklahoma, USA; b St. Vincent’s Institute of Medical Research, Fitzroy, Victoria, Australia; c Victorian Life Sciences Computation Initiative, The University of Melbourne, Carlton, Victoria, Australia; d Data61, CSIRO, Docklands, Victoria, Australia; e Department of Biochemistry and Molecular Biology, Bio21 Molecular Science and Biotechnology Institute, The University of Melbourne, Parkville, Victoria, Australia; f Australian Synchrotron, Clayton, Victoria, Australia; University of Wisconsin-Madison; University of Pittsburgh School of Medicine; University of Alabama at Birmingham

**Keywords:** cold denaturation, complement, gasdermin, perforin, water network

## Abstract

The cholesterol-dependent cytolysin (CDC) genes are present in bacterial species that span terrestrial, vertebrate, and invertebrate niches, which suggests that they have evolved to function under widely different environmental conditions. Using a combination of biophysical and crystallographic approaches, we reveal that the relative stability of an intramolecular interface in the archetype CDC perfringolysin O (PFO) plays a central role in regulating its pore-forming properties. The disruption of this interface allows the formation of the membrane spanning β-barrel pore in all CDCs. We show here that the relative strength of the stabilizing forces at this interface directly impacts the energy barrier posed by the transition state for pore formation, as reflected in the Arrhenius activation energy (E_a_) for pore formation. This change directly impacts the kinetics and temperature dependence of pore formation. We further show that the interface structure in a CDC from a terrestrial species enables it to function efficiently across a wide range of temperatures by minimizing changes in the strength of the transition state barrier to pore formation. These studies establish a paradigm that CDCs, and possibly other β-barrel pore-forming proteins/toxins, can evolve significantly different pore-forming properties by altering the stability of this transitional interface, which impacts the kinetic parameters and temperature dependence of pore formation.

## Abstract (precis)

The cholesterol-dependent cytolysins (CDCs) are the archetype for the superfamily of oligomeric pore-forming proteins that includes the membrane attack complex/perforin (MACPF) family of immune defense proteins and the stonefish venom toxins (SNTX). The CDC/MACPF/SNTX family exhibits a common protein fold, which forms a membrane-spanning β-barrel pore. We show that changing the relative stability of an extensive intramolecular interface within this fold, which is necessarily disrupted to form the large β-barrel pore, dramatically alters the kinetic and temperature-dependent properties of CDC pore formation. These studies show that the CDCs and other members of the CDC/MACPF/SNTX superfamily have the capacity to significantly alter their pore-forming properties to function under widely different environmental conditions encountered by these species.

## INTRODUCTION

**FIG 1:**
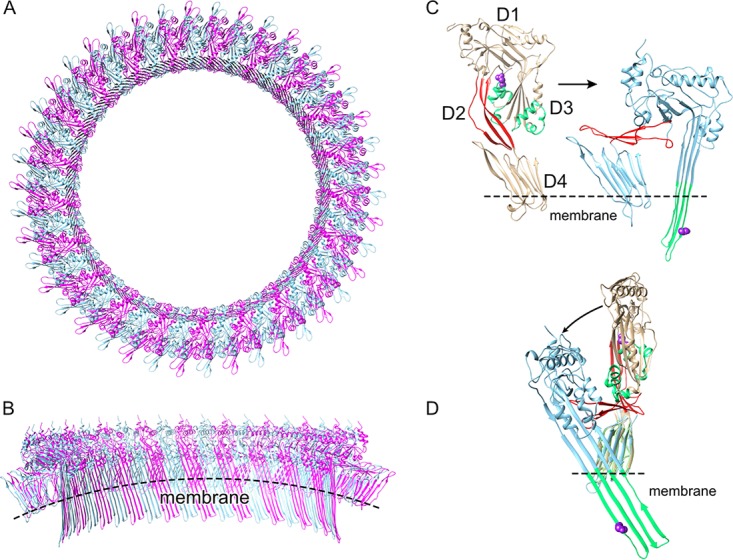
The CDC mechanism. (A) Top-down view of a model of the PFO oligomeric pore complex (monomers shown in alternating red and blue) based on the 4.5-Å resolution cryo-electron microscopy structure of the related CDC, PLY (40). (B) Cutaway view of the pore viewed from inside the lumen. (C) The structure of soluble PFO (22) showing the location of the α-helical bundles (αHBs, green) and the location of N197 (purple space-filled atoms) at the interface between domain 3 and domains 1 and 2 (D3-D1,2). Within the prepore structure, this interface is necessarily disrupted to refold the twin αHBs into the extended transmembrane β-hairpins shown in the structure (right panel) of a PFO monomer extracted from the oligomeric complex, showing that N197 faces the luminal side of the β-barrel in the transmembrane β-hairpin 1 (17, 18). Shown in red in panels C and D is D2, which acts as a ratchet to lower domains D1 and D3 ∼40 Å nearer the membrane so that the extended transmembrane β-hairpins can cross the bilayer to form the β-barrel pore (40–42). (D) Domains D4 of the structures in panel C were overlaid to illustrate the vertical collapse of domains D1 to D3 upon pore formation as viewed from the pore lumen in panel B. Structures were generated using UCSF Chimera (43).

The study of the pore-forming mechanism of the cholesterol-dependent cytolysins (CDCs) has established paradigms that have served as a basis for understanding the mechanisms of a wide array of pore-forming proteins (1), including the membrane attack complex/perforin-like (MACPF) proteins (2) and the stonefish family of venom toxins (SNTX) (3). These proteins all exhibit an analogous protein fold to domain 3 (D3) of the CDCs, which forms the β-barrel pore (Fig. 1C). The species that produce pore-forming proteins of the CDC/MACPF/SNTX superfamily inhabit a variety of environmental niches where temperatures much lower than 37°C are the norm. For instance, the genes for CDCs are present in bacterial species (e.g., Desulfobulbus propionicus [4] and many species of *Paenibacillus* [5], *Bacillus*, and *Lysinibacillus*) which inhabit terrestrial environments at temperatures (6, 7) where CDCs from human pathogens do not function well (8). Therefore, can the CDC mechanism evolve to adapt to function under much different environmental conditions, especially lower temperatures? We have observed that different CDCs exhibit different kinetics and specific activities of pore formation (4, 9, 10), although the basis for these differences has not been explored.

Recently, we showed that an intermolecular electrostatic interaction was necessary to drive the final stage of the prepore-to-pore transition where two α-helical bundles in domain 3 are refolded and extended into the two transmembrane β-hairpins in each monomer of the oligomeric complex to form the large β-barrel pore (11) (Fig. 1 gives an overview of the perfringolysin O [PFO] pore-forming mechanism). We also showed that a point mutant at the interface between domain 3 and domains 1 and 2 of PFO could restore activity to a mutant wherein the electrostatic interaction had been knocked out (loss of the electrostatic interaction traps PFO in a prepore state). These studies suggested that destabilizing this interface structure eliminated the need for the energy supplied by the electrostatic interaction to drive the prepore-to-pore interaction.

In this study, we reveal that the stability of the D3-D1,2 intramolecular interface directly impacts the activation energy of pore formation by PFO. Decreasing the stability of this interface by a point mutation lowers the activation energy of pore formation, which significantly increases the rate and specific activity of PFO-mediated pore formation, and largely eliminates its loss of pore-forming activity at low temperatures. Crystal structures of PFO and this mutant reveal that this interface in PFO is largely stabilized by a water network and its loss in the mutant destabilizes this interface. We further show that the CDC from the terrestrial bacterium *D. propionicus* (desulfolysin [DLY] [4]), unlike PFO, exhibits a high level of pore-forming activity across a wide spectrum of temperatures. Our studies suggest that the ability of DLY to function and remain stable at these widely different temperatures is achieved by balancing polar and nonpolar interactions at analogous interfaces. These studies show that decreasing the stability of this interface in the CDCs can significantly change the activation energy of pore formation, which alters the rate and temperature dependence of pore formation. Hence, the CDC pore-forming structure is sufficiently flexible to adapt it to function under widely different environmental conditions. Since the analogous interface is conserved in the CDC/MACPF/SNTX superfamily (3, 12–16), it is likely the fundamental principles learned here can also be applied their pore-forming mechanisms.

## RESULTS

### Specific activity and temperature dependence of pore formation of various CDCs and their derivatives.

**FIG 2:**
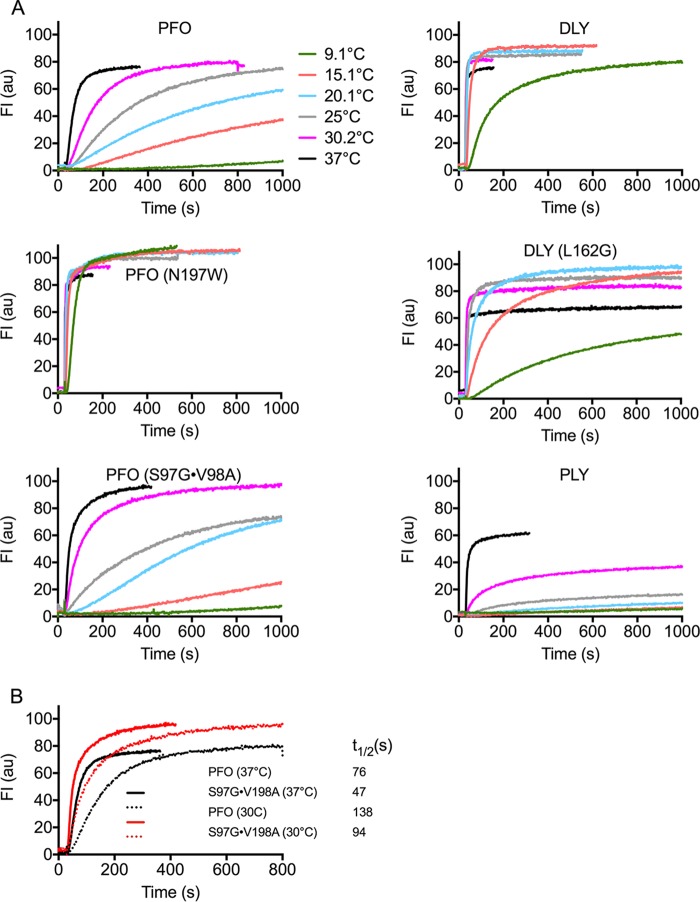
Pore formation rates of CDCs at various temperatures. (A) The rate of pore formation for the indicated CDCs from 9 to 37°C was measured by the release of the fluorescence marker carboxyfluorescein (CF) over time from cholesterol-rich liposomes. Each assay was normalized to the maximum emission obtained with PFO^N197W^ at 37°C. (B) The 30.2°C and 37°C data for PFO and PFO^V97G-S98A^ are overlaid to show that at permissive temperatures the latter exhibits a higher rate of pore formation determined by the time to 50% marker release (*t*_1/2_). Each toxin (36 nM final concentration) was injected into HBS buffer (2 ml) containing carboxyfluorescein (CF)-loaded liposomes (20 μl) at 30 s, and pore formation was monitored by the increase in fluorescence intensity of the CF, as its fluorescence emission was dequenched by release from the liposomes upon pore formation.

The rate of pore formation at temperatures ranging from 9° to 37°C was determined for CDCs from the human pathogens Clostridium perfringens (PFO) and Streptococcus pneumoniae (PLY) and for the terrestrial bacterium *D. propionicus* (DLY) (Fig. 2). For PFO and PLY, the rate of pore formation decreased with decreasing temperature, although PLY activity is much more sensitive to temperatures ≤30°C than PFO. In sharp contrast, pore formation by DLY is much less sensitive to lower temperatures: at 15°C DLY exhibits a similar or higher rate of pore formation than PFO and PLY exhibited at 37°C and at 37°C the specific activity of DLY is nearly 4- to 12-fold higher than that of PFO and PLY, respectively. These results show that DLY-mediated pore formation is faster and far less sensitive to temperature than is PLY- and PFO-mediated pore formation.

We previously described a mutant of PFO (N197W) that compensates for the loss of an electrostatic interaction that drives the prepore-to-pore transition (11). This mutation resides within an intramolecular interface between D3 and D1,2 of the PFO monomer (Fig. 1), which needs to be broken to allow the twin α-helical bundles to refold into extended β-hairpins, which form the β-barrel pore (17, 18). When this mutation is placed into native PFO (PFO^N197W^), its pore-forming parameters resemble those of DLY (Fig. 2 and Table 1). These results and our previous results (11) suggest the impact of the N197W mutation on the PFO pore-forming activity results from perturbations within the D3-D1,2 interface.

**TABLE 1:** Features of PFO and its derivatives^*a*^

Toxin	% activity	*T_m_* (°C)
PFO	100	49.5
PFO^N197W^	380	45.3
PFO^S97G-V98A^	250	50.0
PFO^N197C^	50	48.5
DLY	360	55.9
DLY^L162G^	100	47.0
PLY	28	48.9

a Shown are the relative pore-forming activities of the various CDCs in this study and their melting temperatures (*T_m_*). The pore-forming activity for each CDC and derivatives thereof relative to the pore-forming activity of wild-type PFO was determined from the EC_50_ of pore formation on carboxyfluorescein-loaded cholesterol-rich liposomes (4) and reported as the percentage of PFO activity at 37°C [% activity = (EC_50_ PFO/EC_50_ mutant) × 100]. The results are representative of 3 to 4 experiments.

### Structural transitions associated with pore formation are accelerated in PFO^N197W^.

To understand if one or more of the structural transitions necessary for pore formation were affected by the N197W mutation, we used previously established fluorescence-based methods to compare the rate of these transitions to those in PFO.

**FIG 3:**
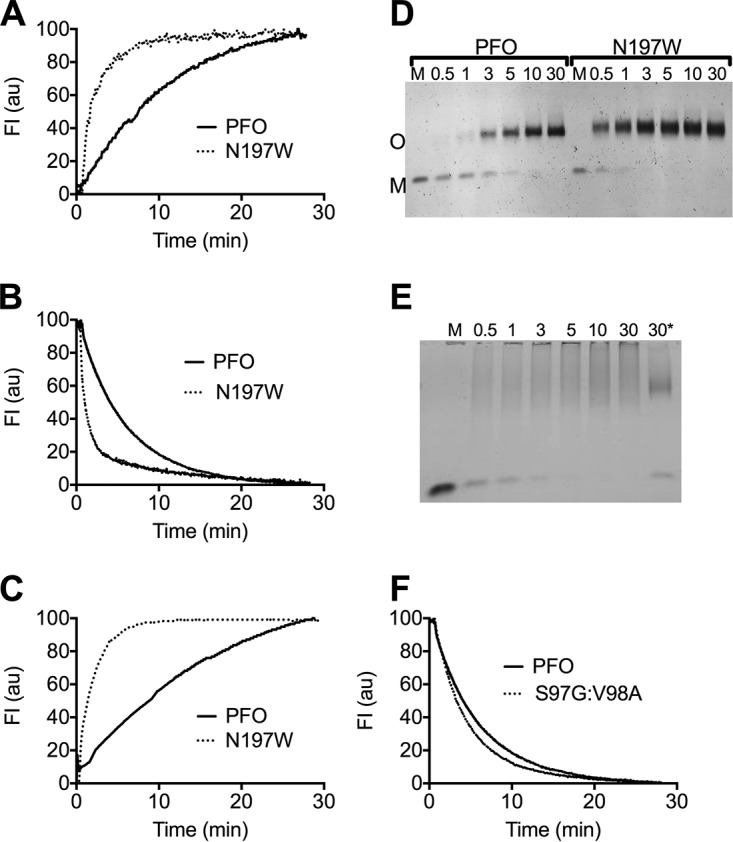
Structural transitions required for oligomerization and pore formation are accelerated in PFO^N197W^. (A) The rate of membrane binding was determined by following the increase in the emission over time of the UDP tryptophan residues in D4 (19), as monomers of PFO (solid line) and PFO^N197W^ (dashed line) bind to the liposomes. (B) The rate of monomer oligomerization into the prepore complex was determined by following the disengagement of β5 from β4. This disengagement was detected by monitoring the decrease in the emission over time of NBD-modified cysteine-substituted V322 in PFO^V322C^ (solid line) or PFO^V322C-N197W^ (dashed line). V322 is buried beneath the α1β5 loop (Fig. 4A, left panel), and upon the disengagement of β5 from β4 during oligomerization, NBD is exposed to water, which quenches its emission (20). (C) The membrane insertion of the β-barrel was determined by the increase in the fluorescence emission of NBD positioned on cysteine-substituted A215 (18), a membrane-facing residue in transmembrane β-hairpin 1, in PFO^A215C^ (solid line) and PFO^A215C-N197W^ (dashed line). All data were normalized to 100 and are representative of 3 independent experiments. (D and E) PFO, PFO^N197W^, or DLY was injected into buffer containing cholesterol-rich liposomes, and samples were withdrawn at the times indicated and immediately placed into SDS-PAGE sample buffer to quench the assembly of the pore. The samples were then separated by SDS-AGE to separate the monomer (M) from the oligomer (O). Note that the disappearance of the DLY monomer is rapid, as it assembles into the pore complex, which tends to form a smear in the absence of heat. *, same as the DLY 30-min sample that was additionally heated to 95°C for 3 min to show that the smeared oligomers migrate as a single band when heat is applied. (F) The rate of the disengagement of β5 from β4 in PFO^S97G-V98A^ compared to PFO was measured as described in the legend to panel B. All fluorescence assays were carried out at 37°C. M, soluble toxin monomer in the absence of liposomes.

**FIG 4:**
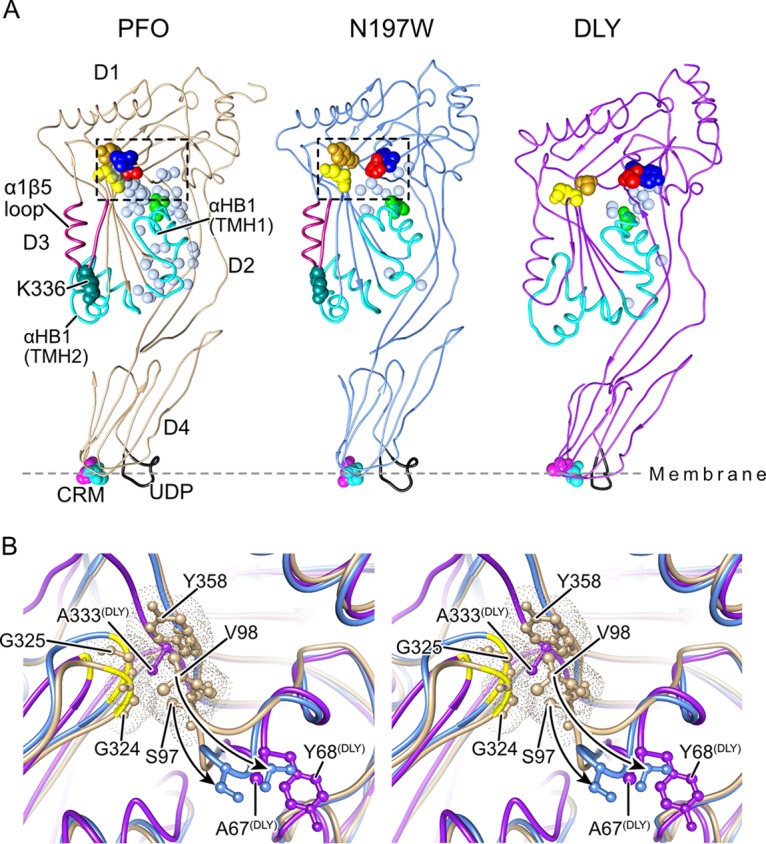
The solution of the crystal structures of DLY and PFO^N197W^ reveals differences with PFO at the D3-D1,2 interface. (A) The α-carbon backbone representation of the crystal structures of the previously solved structure of PFO (22) and the structures solved here for PFO^N197W^ and DLY. The waters at the D3-D1,2 interface are shown for all three proteins as light blue transparent Van der Waals (VDW) representations. Also shown are the conserved diglycine pairs in D3 (yellow space-filled atoms), the S97 and V98 pair (red and blue space-filled atoms, respectively), Y358 (gold space-filled atoms), the α-helical bundles (αHBs) that ultimately refold to form transmembrane β-hairpins 1 and 2 (cyan), the α1β5 loop (dark red), and the structures of the membrane binding interface: the cholesterol recognition/binding threonine-leucine pair (CRM) and the undecapeptide (UDP, shown in black). (B) Stereo image showing the region boxed in PFO (tan) in panel A overlaid with the same regions in PFO^N197W^ (light blue) and DLY (purple), showing the displacement of the D96-Y103 loop (shown by the arrows) in PFO^N197W^ and specifically showing the displacement of S97 and V98 from their interaction with the conserved glycine pair (G324, G325), which serves as a flexible linker to allow the rotation of β5 away from β4 (20) and from Y358. The positions of the analogous residues (A67 and Y68) are shown for DLY, and the figure shows that the analogous residue for Y358 in PFO is an alanine in DLY.

Membrane binding kinetics of PFO and its derivatives were measured by following the increase in the fluorescence emission of the tryptophans within the conserved undecapeptide, as it enters the membrane upon binding (19). PFO^N197W^ binding is ∼4-fold faster than PFO (Fig. 3A). Since this mutation is distal from the binding domain, it does not impact the binding interface structure (as confirmed below in the PFO^N197W^ crystal structure); therefore, the initial interaction of the monomers with cholesterol would not be affected. An alternative mechanism to account for the high membrane affinity of PFO^N197W^ would be a faster incorporation of monomers into the higher-avidity prepore complex (8), thereby decreasing the off-rate of membrane-bound monomers. This scenario was confirmed by the higher rate of oligomerization for PFO^N197W^ than PFO. Oligomerization drives the disengagement of β-strand 5 (β5) from β-strand 4 (β4), which facilitates the formation of stable prepore oligomers (11, 20, 21). Thus, the rate at which β5 disengages can be used to monitor oligomerization. The rate of β5 disengagement was followed by monitoring the change in fluorescence emission of the environmentally sensitive probe NBD attached to V322C, as it makes the transition from a buried location under the α1β5 hairpin (Fig. 4A) in the soluble monomer to a solvent-exposed location in the oligomer where its emission is quenched by water. The disengagement of β5 from β4 is ∼5-fold faster in PFO^N197W^ than for PFO (Fig. 3B). Oligomerization rates for PFO and PFO^N197W^ (Fig. 3D) were also determined by SDS-agarose gel electrophoresis (SDS-AGE) of samples removed at various times from toxin-liposomes mixtures to assess formation of the oligomer (Fig. 3D). DLY oligomerization was also measured by SDS-AGE as for PFO and PFO^N197W^, which showed it oligomerized faster than PFO (Fig. 3E).

The changes in PFO^N197W^ pore-forming properties come at the cost of decreased stability at higher temperatures. Whereas PFO retains full pore-forming activity and oligomer formation on liposomes after being incubated at 45°C prior to the addition of liposomes, PFO^N197W^ completely lost the capacity to form oligomers and pores at 45°C (see Fig. S1 in the supplemental material). This is consistent with the reduced thermal stability (see Materials and Methods) of PFO^N197W^ (Table 1), which is highly dependent on the D3-D1,2 interface (11).

10.1128/mBio.00538-19.1 FIG S1 PFO and PFO^N197W^ oligomer and pore formation sensitivity to elevated temperature. Download FIG S1, DOCX file, 0.2 MB. Copyright © 2019 Wade et al. 2019 Wade et al. This content is distributed under the terms of the Creative Commons Attribution 4.0 International license.

Upon completion of the prepore complex, the D3-D1,2 interface in each monomer is disrupted to extend ɑ-helical bundle 1, which along with ɑ-helical bundle 2 refolds into transmembrane β-hairpin 1 and transmembrane β-hairpin 2, respectively, to form the β-barrel pore (Fig. 1) (17, 18). The insertion of the β-barrel, as measured by the increase in the fluorescence emission of NBD-labeled A215C in transmembrane β-hairpin 1, as it transitions from a polar environment in the soluble monomer to the nonpolar environment of the membrane (18), was ∼6-fold faster in PFO^N197W^ than PFO (Fig. 3C). Hence, all measurable transitions, from binding to the insertion of the β-barrel pore, were accelerated in PFO^N197W^.

### Crystal structures of PFO^N197W^ and DLY.

To gain insight into the structural basis for the differences in pore-forming activity of PFO versus that of PFO^N197W^ and DLY, we solved the crystal structures for the latter two proteins (Fig. 4A). Two crystal forms for PFO^N197W^ were generated: one diffracted to 3.3-Å resolution (PFO^N197Wlow^) and the other diffracted to 2.7-Å resolution (PFO^N197Whigh^) (Table S1). The electron density maps of PFO^N197Whigh^ were of sufficient quality to identify 101 water molecules. Most of the following analyses focus on comparison of the wild-type structure of PFO (22) and that of DLY with PFO^N197Whigh^, but we have also compared them to the PFO^N197Wlow^ structure, where differences with native PFO might be due to crystal lattice effects.

10.1128/mBio.00538-19.4 TABLE S1 Crystallographic data and refinement statistics. Crystal structures of PFO^N197W^ and DLY. Download Table S1, DOCX file, 0.02 MB. Copyright © 2019 Wade et al. 2019 Wade et al. This content is distributed under the terms of the Creative Commons Attribution 4.0 International license.

DLY was crystallized, and its structure was determined by multiple anomalous dispersion to a resolution of 2.3 Å (Table S1). Despite the relatively low sequence homology between DLY and CDCs from Gram-positive bacteria (∼40% identity with PFO) with known crystal structures, DLY adopts a similar overall shape, topology, and domain arrangement (Fig. 4A). Most CDCs have just one cysteine residue, which is located in the undecapeptide motif, whereas DLY has three additional cysteine residues (C54 in D2 and C166 and C266 in D3), none of which are in a disulfide, although C166 could not be altered without decreasing pore-forming activity (E. M. Hotze and R. K. Tweten, unpublished data; also reference 4).

### PFO^N197W^ and DLY D3-D1,2 interface structures.

The oligomerization and assembly of the membrane-spanning β-barrel pore require the twist in the D3 core β-sheet to be relieved (Fig. 1) to pair β-strands 1 and 4 between two monomers (20). The next step is that the D3-D1,2 interface of each monomer in the completed prepore needs to be disrupted to refold and extend αHB1 into one of the two transmembrane β-hairpins to form the large β-barrel pore (Fig. 1) (17, 18). In PFO, the majority of the interactions at this interface in the soluble monomer are mediated by an extensive water network (23), which is largely missing in the vicinity of N197W (Fig. 4 and Fig. S2), although care must be taken in comparing the fine detail of the two structures given their different resolutions. The bulkier side chain of the tryptophan residue clearly displaces two waters observed in PFO (waters 584 and 756 using 1PFO numbering), and the movement of the side chain of N377 displaces water 704 (Fig. S2). Loss of these three waters is expected to disrupt the local H-bonding network around N197W. In PFO, N197 forms hydrogen bonds (H-bonds) with waters 657 and 795. The former water is not in H-bonding distance of any other waters but is close to water 683 (3.3 Å). The latter water is within H-bonding distance of waters 704 (lost by the movement of N377 in the mutant) and 756 (a water displaced by the mutation). Thus, in PFO^N197W^ this water has lost all its H-bonding partners observed in PFO, which provides an explanation for its absence in the mutant.

10.1128/mBio.00538-19.2 FIG S2 Environment of N197 and N197W. Download FIG S2, DOCX file, 0.1 MB. Copyright © 2019 Wade et al. 2019 Wade et al. This content is distributed under the terms of the Creative Commons Attribution 4.0 International license.

To overcome the uncertainty resulting from the difference in resolution of the crystallographic structures of PFO and PFO^N197W^ and the consequent reduction in visible waters in PFO^N197W^, we carried out molecular dynamics (MD) simulations to explore differences in the dynamic behavior of the D3-D1,2 interface. The stability of water molecules around residue 197 was determined by creating a solvent residence map across the simulation of each protein (24). The stable solvent includes several of the crystallographic waters seen in PFO. There are two regions of stable solvent in the PFO simulation capping the C-terminal end of the D3 helix (residues 188 to 197) at residues 194 and 195 that are directly disrupted in PFO^N197W^, with the tryptophan displacing all solvent in this volume (Fig. S3A). Its side chain also disrupts the stable water volume associated with the backbone of residue 196 through hydrophobic effects. This alteration in the solvation cap around the C-terminal end of the helix is consistent with a loss of stability around residue 197.

10.1128/mBio.00538-19.3 FIG S3 MD simulations showing the impact of the PFO^N197W^ mutation on the D3-D1,2 interface waters. Download FIG S3, DOCX file, 2.3 MB. Copyright © 2019 Wade et al. 2019 Wade et al. This content is distributed under the terms of the Creative Commons Attribution 4.0 International license.

The simulations also revealed a stable water chain reaching from the side chain of N197 to a position between the backbone carbonyl oxygens of S93 and L114, corresponding to crystallographic waters 683, 581, and 637 in 1PFO. This chain is broken in the simulations of PFO^N197W^, with no equivalent stable waters (Fig. S3B). The presence of the stable water chain in PFO and its absence in PFO^N197W^ are consistent with the dependence of the D96-Y103 interaction with the β4-β5 loop glycine pair (G324, G325) on water-mediated interactions in this region.

The D3-D1,2 interface in DLY differs substantially from that in PFO. There are fewer than half as many D3-D1,2 interfacial water molecules identifiable in the crystal structure of DLY, compared to PFO (15 and 37, respectively), which can be attributed, in part, to the greater number of bulky nonpolar residues that result in a more tightly packed interface that excludes solvent. In DLY, 49% of the interfacial residues are nonpolar compared to 38% in PFO, and to a large extent, the nonpolar residues in PFO are not in complementary interactions (i.e., hydrophobic-hydrophobic) (22), whereas they are in DLY. Consequently, there is a greatly reduced number of hydrogen bonding interactions present across the interface, with 12 protein-to-protein H-bonds in PFO compared to only 6 in DLY. Bridging waters (which form H-bonds with residues in both D3 and D1,2) are also reduced from 12 in PFO to 7 in DLY.

The only significant deviation between the PFO and PFO^N197W^ is a loop that is displaced 7 Å in the latter and encompasses D1 residues D96 to Y103 (Fig. 4B). This difference partially results from contacts with the N-terminal helix of a symmetry-related molecule in the crystal lattice of both mutant structures, which are not seen in PFO. However, other PFO crystal structures (23) exhibit a similar packing arrangement in this region but do not show the displacement of this loop, which strongly suggests that the loop is specifically displaced in PFO^N197W^ independently of the crystal contacts. Interestingly, the analogous loop in native DLY (S66 to P74) adopts a conformation similar to that observed in PFO^N197W^ where it does not contact the glycine pair analogue (G296, G297) in DLY (Fig. 4B). The glycine pair is a flexible linker region that allows the rotation of β5 away from β4, which allows the subsequent edge-on pairing of β4 of one monomer with β1 of another monomer during their oligomerization (20). These results suggest that the conformational differences in the D3-D1,2 interface between PFO and that of DLY and PFO^N197W^ contribute to the difference in the activity and the sensitivity of pore formation to low temperatures.

We mimicked the loss of the contact of the D96 to Y103 loop with the loop containing the conserved glycine pair by removing the S97 and V98 side chains in PFO^S97G-V98A^. Its specific activity (Table 1) and rate of pore formation (Fig. 2B) increased, but it did not alter the temperature dependence of pore formation (Fig. 2A), consistent with the similarity of its melting temperature (*T_m_*) to that of PFO (Table 1). The rate of β5 disengagement from β4, which reflects the rate of oligomerization of the monomers into the prepore (20), was only slightly higher than PFO (Fig. 3F). These results suggest the primary impact of rotating this loop away from the glycine pair is to increase the rate of the prepore-to-the-pore transition, which suggests that it is a necessary conformational change for the step in pore formation wherein the prepore is converted to the pore. The mutation, however, had no discernible effect on the temperature dependence of pore formation. Hence, the stability of the D3-D1,2 interface is the major factor affecting the temperatures dependence of PFO.

### Chemical modification of the PFO^N197C^ sulfhydryl triggers its transition to the pore at low temperature.

**FIG 5:**
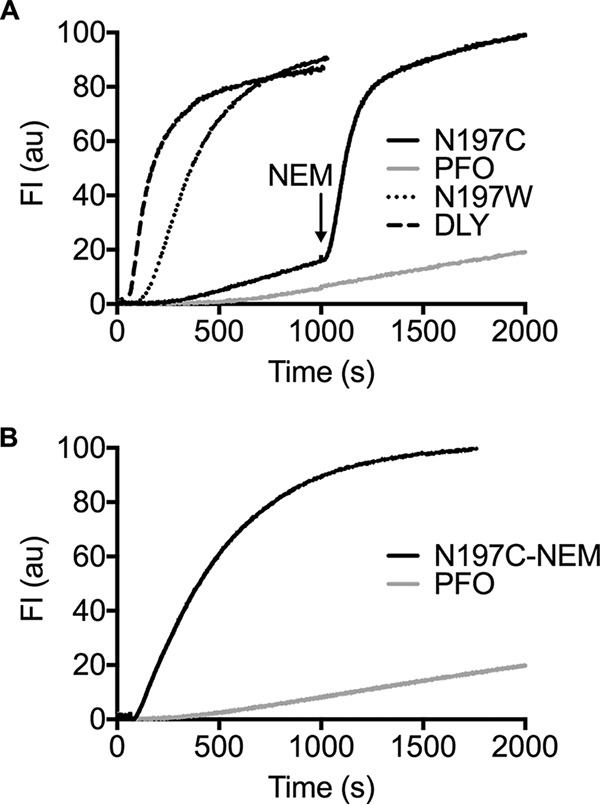
Pore formation by PFO^N197C^ at low temperature modified with NEM before and after prepore assembly. (A) Pore formation was monitored over time by CF release from liposomes treated with PFO (±NEM), PFO^N197W^, PFO^N197C^ (±NEM), and DLY at 6°C. CF release by PFO and PFO^N197C^ was monitored for 1,000 s to allow for prepore formation, and then *N*-ethylmaleimide (NEM) was injected (final concentration of 100 μM) to modify the sulfhydryl of N197C. As shown, NEM triggers the rapid conversion of prepore PFO^N197C^ but has no effect on the low rate of pore formation by PFO. (B) The cysteine sulfhydryl of PFO^N197C^ was labeled with NEM prior to injecting it into liposomes at 6°C compared to PFO. Note that the rate of pore formation by PFO^N197C^ is higher when the prepore is allowed to first assemble followed by the injection of NEM in panel A than when PFO^N197C^ is labeled with NEM prior to its injection into the liposomes in panel B.

Like PFO (8), low temperature significantly slows the conversion of the PFO^N197C^ prepore to the pore, although it converts to a pore state at a slightly higher rate than PFO. This suggests that the water network is largely intact in the D3-D1,2 interface of PFO^N197C^, which is consistent with the similarity of its *T_m_* (48.5°C) to that of PFO (49.5°C). When PFO^N197C^ was allowed to first assemble into a prepore at low temperature, its transition to the pore could be rapidly triggered by the injection of *N*-ethylmaleimide (NEM), a small hydrophobic compound that modifies cysteine thiols (Fig. 5), whereas NEM had no effect on native PFO. PFO^N197C^ labeled with NEM prior to its injection exhibited similar pore-forming activity at low temperature (Fig. 5B), but the rate was slightly lower than that observed in Fig. 5A, where it was first allowed to form the prepore for the addition of the NEM. Therefore, the water network in PFO^N197C^ still maintained the prepore state until NEM modified the N197C thiol. Hence, the modification of the thiol with NEM likely mimics the effect of the substitution of tryptophan for N197 by displacing the stabilizing water network at the D3-D1,2 interface, which can be effectively accomplished before or after prepore assembly.

### Arrhenius activation energy of pore formation.

**FIG 6:**
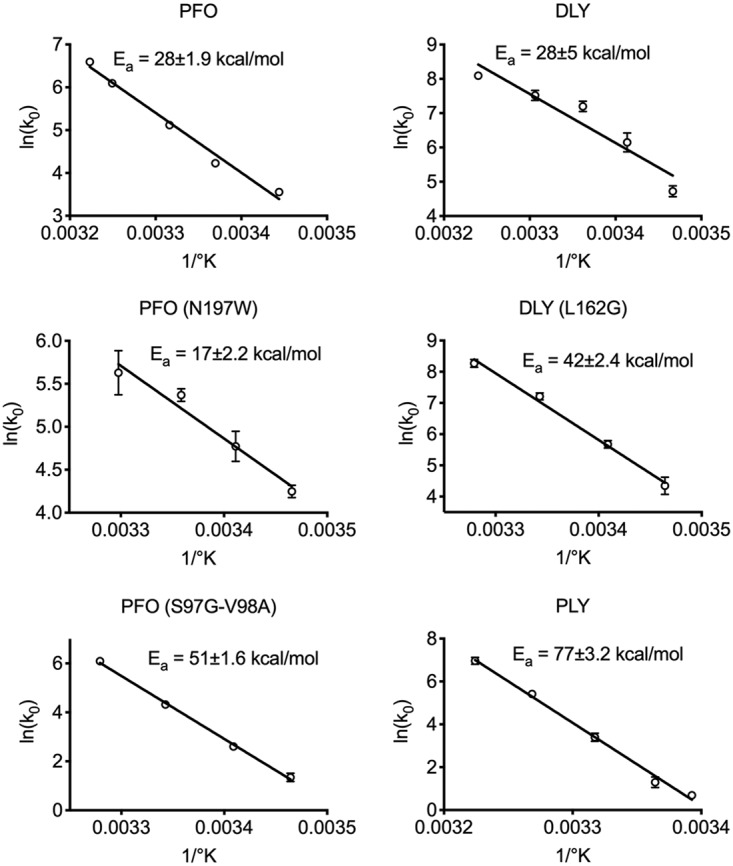
Arrhenius activation energies of pore formation by PFO, DLY, and their derivatives. The Arrhenius activation energies (E_a_) were determined for the proteins shown. The initial velocities (*k*_0_) of pore formation (the linear portion of the marker release curve, as in Fig. 2) at the various temperatures were used to determine the E_a_. Each analysis was carried out in quadruplicate, and the standard error (SE) for 1/*k*_0_ at each temperature is shown. Those SEs with error bars smaller than the symbols are not visible.

We next examined the Arrhenius activation energies (E_a_) of pore formation by the various CDCs and derivatives (Fig. 6). Since PFO^N197W^ presumably lost most of its D3-D1,2-stabilizing water network, we hypothesized that its E_a_ would decrease substantially, thereby explaining the increased rate of pore formation and activity at low temperature. The initial velocities of pore formation by each toxin were measured across a range of temperatures, and this information was then used to calculate the E_a_. Consistent with the above hypothesis, PFO^N197W^ exhibited an E_a_ of 17 kcal/mol, which is ∼10 kcal/mol lower than that for PFO and DLY (∼27 kcal/mol each). PLY exhibits a significantly higher E_a_ (77 kcal/mol) than both PFO and DLY, which correlates with weak pore-forming activity at temperatures below 37°C (Fig. 2A).

These data suggest that the loss of the D3-D1,2-stabilizing water network in PFO^N197W^ reduces its thermal stability, lowers its E_a_ for pore formation, and increases the specific activity of pore formation and pore formation at lower temperatures. These improved pore-forming parameters in PFO^N197W^, however, come at a cost: They reduce protein stability at higher temperatures. The fact that DLY exhibits the same E_a_ as PFO but has a higher thermal stability suggests that DLY has evolved a different way to function at low temperature while remaining stable at higher temperatures.

### The DLY D3-D1,2 residue L162 side chain-mediated interactions impact its pore-forming activity and temperature dependence of pore formation.

**FIG 7:**
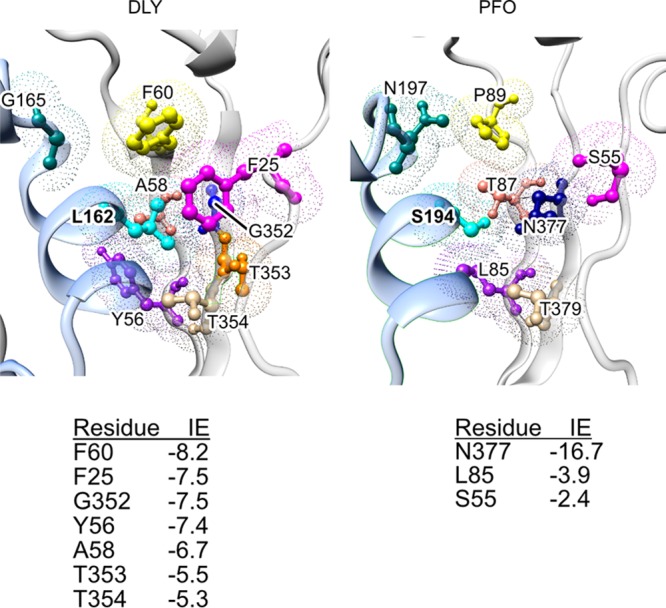
DLY D3-D1,2 interface near L162. Leucine 162 of DLY is located in a hydrophobic pocket primarily composed of aromatic residues F25, Y56, F60, and A58, whereas the analogous residues in PFO are primarily hydrophilic residues. Also shown is the location of N197 in PFO, which has been replaced by G165 in DLY. The interaction energies (IE) (http://bioinfo.uochb.cas.cz/INTAA/energy/) of the major residues that interact with DLY L162 and its analog in PFO, S194, are shown for each protein.

DLY has a 6°C-higher *T_m_* than PFO (Table 1), suggesting that its D3-D1,2 interface is more stable than that of PFO, yet it functions much better at low temperature than PFO (Fig. 2). One significant difference in the D3-D1,2 interface with that of PFO is the presence of a strong nonpolar interaction between the side chain of L162 in αHB1 (which refolds into transmembrane β-hairpin 1) and a hydrophobic core of D2 residues consisting of F25, Y56, A58, and F60 (Fig. 7). When L162 was changed to a glycine, its *T_m_* dropped nearly 9°C (Table 1), showing that it is a major contributor to the stability of the D3-D1,2 interface. The specific activity of DLY^L162G^ decreased nearly 4-fold, consistent with its higher E_a_ of its pore formation of 42 kcal/mol (Fig. 6), compared to DLY. The impact of weakening the D3-D1,2 interface on the specific activity of DLY and temperature dependence of pore formation was surprising compared to PFO^N197W^, which exhibited enhanced pore-forming activity across the temperature spectrum. These results suggest that the L162-mediated nonpolar interactions with D2 residues positively impact its activity at the low and high end of the temperature range.

## DISCUSSION

The studies here show that the relative stability of the D3-D1,2 interface has a major impact on the transition state for pore formation by PFO. The stability of this interface affects the E_a_ of pore formation, which impacts all stages of the pore-forming mechanism. All CDCs have the analogous interface structure; thus, the relative stability of this interface impacts the pore-forming activity of all CDCs. In PFO, this interface is primarily stabilized by a network of water-mediated H bonds, and its disruption in PFO^N197W^ dramatically improves the pore-forming properties of PFO and largely eliminates its loss of activity at low temperatures where it is normally trapped in a prepore complex (8). Native DLY exhibits similar pore-forming properties as PFO^N197W^, which is consistent with the adaptation of the DLY structure and mechanism to function under the wide range of temperatures which are encountered by *D. propionicus* in its terrestrial niches. The studies here, however, suggest that DLY has evolved a different D3-D1,2 structure to maintain a high level of pore-forming activity and stability over a broad range of temperatures. Rather than being dominated by an H-bonded water-stabilized interface like PFO, the DLY D3-D1,2 interface is stabilized by a mix of polar and nonpolar interactions. Polar and nonpolar interactions weaken and strengthen inversely with temperature, which would tend to minimize changes in the strength of the interactions that stabilize the DLY D3-D1,2 interface across a wider range of temperatures. This would minimize changes in the strength of the transition state for pore formation, thereby ensuring that DLY remained active and stable across a wider range of temperatures.

The crystal structure of PFO^N197W^ and MD simulations show that the water network throughout the D3-D1,2 interface of PFO is largely disrupted, consistent with its lower *T_m_*. The weakened D3-D1,2 interface lowers the E_a_ for pore formation, which results in faster kinetics of prepore assembly and conversion to the pore and alleviates the loss of activity at lower temperatures. This scenario is further supported by the experiments with PFO^N197C^ wherein the low-temperature-trapped prepore could be triggered to rapidly convert to the pore by the NEM modification of the cysteine thiol. The hydrophobic nature of NEM likely disrupted the water network by the steric/hydrophobic displacement of the waters in a manner similar to that of the hydrophobic side chain of tryptophan in PFO^N197W^. Hence, in PFO as the temperature decreases, the water-mediated H bonds at this interface increase in strength, which effectively increases the energy barrier of the transition state to a point where the forces normally driving this change are no longer sufficient. The loss of the water network in PFO^N197W^ effectively diminishes these stabilizing forces at the D3-D1,2 interface. Therefore, the forces that drive the assembly of the pore remain sufficient to overcome the transition state at the lower temperatures.

In contrast to PFO, DLY exhibits significant pore-forming activity between 15 and 37°C, yet it exhibits a significantly higher *T_m_* than PFO (∼6°C) and the same E_a_. The higher *T_m_* is consistent with the presence of more nonpolar interactions at its D3-D1,2 interface, which increase in strength with increasing temperature (25). L162 mediates a major nonpolar interaction between D3 and D2 (Fig. 7). Although the rate of pore formation by DLY^L162G^ increased with temperature, it released less total CF marker below 20°C and above 25°C than DLY, but at 20 to 25°C total marker release was similar to DLY. At lower temperatures, nonpolar interactions weaken with decreasing temperature and have been shown to be responsible for cold denaturation of proteins at temperatures above 0°C in the absence of denaturants (26–29). Hence, at lower temperatures it is possible that the destabilizing effect of this nonpolar interaction is lost in DLY^L162G^, which would make it more difficult to disrupt its domain 3-domain 1,2 interface and convert to the pore. Above 25°C, the loss of the L162-mediated hydrophobic interactions would result in a less stable D3-D1,2 interface, which is consistent with its lower *T_m_* and may lead to the premature unfolding and inactivation of DLY monomers. Therefore, we suggest that in native DLY the opposing effects of the polar and nonpolar interactions with temperature would minimize changes in the overall stability of the D3-D1,2 interface, which allows the forces driving this transition to be adequate across a broad range of temperatures.

Collectively, these data show that the loss of the water network that stabilizes the D3-D1,2 interface in PFO has far-reaching effects on all aspects of its pore-forming mechanism. The increased rate of oligomerization suggests that the weakened D3-D1,2 interface must facilitate early conformational changes necessary for the stable interaction of membrane-bound monomers. The increased rate of monomer incorporation into the higher-avidity prepore complexes effectively decreases the monomer off-rate from the membrane, which lowers the EC_50_ of pore formation. The disruption of the S97-V98 loop interaction with the G324-G325-containing loop contributes to the increased rate of pore formation but not the temperature dependence of PFO. The network of water-mediated H bonds at the D3-D1,2 interface in PFO is the dominant factor responsible for the loss of pore-forming activity as the temperature decreases. In contrast to PFO, DLY has evolved a different solution to function and remain stable across a wide range of temperatures by including a combination of both polar and nonpolar interactions at its D3-D1,2 interface. Finally, PFO^N197W^ demonstrates that the CDC pore-forming mechanism is capable of maintaining high activity down to 6°C, and probably lower. This shows that the CDC structure can evolve to function well at extremely low temperatures, which may be necessary for CDC-expressing species that are found in terrestrial habitats where temperatures remain near freezing much of the year (6, 7).

The temperature dependence of the CDCs here reflects the lifestyles of the bacterial species. *D. propionicus* exhibits growth across a broad range of temperatures (10 to 40°C) (30), which correlates with the temperature range at which DLY retains significant activity. C. perfringens is present in terrestrial environments and intestinal tracts of humans and animals; however, its growth decreases rapidly at temperatures of ≤30°C (31, 32), which corresponds to the temperature dependence of PFO pore-forming activity. In contrast to PFO and DLY, PLY pore formation is most efficient at 37°C and drops off rapidly below 37°C, which is consistent with its evolution as a human commensal. PLY is unusual in that it appears to be secreted by an unknown mechanism and is retained for long periods within the cell wall of S. pneumoniae (33). In its commensal state, the higher E_a_ may minimize the possible activation of PLY (even in the absence of cholesterol) where it could damage the bacterial cell.

These studies show that the evolution of the D3-D1,2 interface structure can endow the CDCs with significantly different pore-forming properties. The modulation of these properties likely accommodates the requirements of the myriad species that utilize CDCs to establish and/or maintain their presence in their respective niches.

## MATERIALS AND METHODS

### Mutagenesis, expression, and purification of PFO, DLY, and their derivatives.

*In vitro* mutagenesis of the codon-optimized DLY and PFO genes, their expression, and purification of the recombinant proteins and derivatives from Escherichia coli Tuner were carried out as previously described (11, 18). The cysteine-less derivative of PFO (PFO^C459A^) is referred to here as *PFO*. All proteins and chemically modified proteins were centrifuged (14,000 × *g* for 10 min at 4°C) to remove any precipitated protein and assayed for concentration before use as described previously (10). Selenomethionine derivatives of DLY were obtained by expression using the selenomethionine kit from Molecular Dimensions (USA), according to the manufacturer’s instructions.

### Liposome preparation.

Liposomes that contained a 45:55 mol% ratio of 1-palmitoyl-2-oleoyl-*sn*-glycero-3-phosphocholine (Avanti Polar Lipids) to cholesterol (Sigma-Aldrich) were prepared as described previously (18). The same liposomes with encapsulated 5(6)-carboxyfluorescein (CF) (Sigma) were prepared as described previously (4).

### Carboxyfluorescein-liposome release assay for the EC_50_ analysis.

The EC_50_, which is the effective concentration of toxin required to release 50% of the encapsulated CF from liposomes, was measured and calculated as described previously (4).

### Modification of PFO with fluorescent probes.

PFO derivatives with a cysteine residue substituted at various positions were modified via the sulfhydryl group with the sulfhydryl-specific fluorescent dyes IANBD [*N*,*N*′-dimethyl-*N*-(iodoacetyl)-*N*′-(7-nitrobenz-2-oxa-1,3-diazol-4-yl)ethylenediamine], Alexa Fluor 488 C_5_ maleimide, and tetramethylrhodamine-5-maleimide (ThermoFisher), as previously described (20).

### SDS-agarose gel electrophoresis (SDS-AGE).

Oligomer formation by PFO and its derivatives was determined by SDS-AGE as previously described (8).

### PFO, DLY, and derivative melting temperatures.

The *T_m_* was determined as previously described (11). Briefly, the *T_m_* of the proteins was determined using a 7500 Fast real-time PCR system to generate the thermal gradient. Unfolding of the proteins was detected using the Protein Thermal Shift Dye kit (Applied Biosystems) according to the manufacturer’s protocol wherein the fluorescent dye binds to exposed hydrophobic regions of the proteins as it unfolds. The *T_m_* represents the temperature at which 50% unfolding is detected.

### Fluorescence spectroscopic methods.

All fluorescence measurements (except for the *T_m_* determination) were performed on a Fluorolog-3-Spectrofluorometer with FluorEssence software (Horiba Jobin Yvon) or on an SLM 8100 fluorimeter. The excitation and emission wavelengths for 5(6)-carboxyfluorescein (CF), NBD, and tryptophan were 480 and 520 nm, 478 and 530 nm, and 280 and 340 nm, respectively.

The kinetics of pore formation by PFO and its derivatives were determined by using CF-loaded liposomes as described previously (11). The fluorescence emission of CF concentrated in the liposomes is quenched, and upon pore formation, the release of the CF results in dequenching of its emission due to dilution. The emission increase as the CF was released was monitored over time.

The kinetics of PFO and PFO^N197W^ binding to liposome membranes were measured by following the emission of the 3 tryptophans in the undecapeptide (UDP), which insert into the membrane upon binding (19). Upon membrane binding, the UDP tryptophans transition from the polar environment in the soluble monomer and partition into the nonpolar environment of the bilayer, which results in the increase of their emission at 340 nm, as they are no longer quenched by water.

The kinetics of β-barrel insertion were determined as previously described (19). Residue A215 was substituted with cysteine in PFO and PFO^N197W^. A215 partitions its side chain into the bilayer upon formation of the β-barrel pore and has been used extensively by us to monitor the kinetics of β-barrel insertion. The A215C was modified with the environmentally sensitive probe *N*,*N*′-dimethyl-*N*-(iodoacetyl)-*N*′-(7-nitrobenz-2-oxa-1,3-diazol-4-yl)ethylenediamine (IANBD, or NBD here). Upon the transition from the polar environment of the soluble PFO monomer, wherein water quenches the emission of the NBD, to the insertion of the β-barrel pore, the NBD emission increases due to the exclusion of water.

To measure the oligomerization of PFO or PFO^N197W^, each was substituted with V322C, which was then labeled with NBD. Upon oligomerization, V322 undergoes a nonpolar-to-polar transition (measured as a decrease in the emission of NBD) as the α1β5 loop rotates away from β4, thereby exposing the edge of β4 (20). The exposure of β4 of one monomer then allows it to pair and form backbone H bonds with β1 of another monomer during oligomerization of the prepore.

For the membrane insertion and oligomerization assays, self-quenching of the NBD due to proximity in the oligomer was minimized by mixing a 1:2 ratio of NBD-labeled protein with unlabeled protein. For the membrane binding, membrane insertion, and oligomerization assays, the final concentration of toxin was 7 nM in a 2-ml volume containing 20 μl of liposomes from a stock prepared as described above. Toxin was injected (50 μl) into the cuvette (2 ml HEPES-buffered saline [HBS] containing 20 μl liposomes) at 30 s, and the emission was followed over time.

### Arrhenius energy of activation (E_a_) determination.

The E_a_ for CDC-mediated pore formation was calculated as described previously (11). The initial velocities of pore formation at various temperatures were determined in quadruplicate and used to calculate the E_a_ for PFO, DLY, PLY, and their derivatives. Initial velocities were derived from the linear portion of the CF release curve. Each cuvette contained 2 ml HBS (20 mM HEPES-buffered saline, pH 7.4) into which 50 μl of each CDC was injected to a final concentration of 37 nM for PFO, 56 nM for PLY, and 3.7nM for DLY and PFO^N197W^ (due to the difference in specific activities of each toxin). Temperature was controlled using a recirculating chiller that maintained the fluorimeter cuvette turret at the selected temperature.

### Crystallization of PFO^N197W^ and DLY.

Crystals were obtained from PFO^N197W^ (5 mg/ml) using the hanging drop method with precipitant of 9% (wt/wt) PEG 6000, 100 mM HEPES, pH 7.0, and 2% (vol/vol) dioxane at 21°C. Crystals of DLY (2.6 mg/ml, 20 mM Tris, pH 7.2, 500 mM NaCl, 5 mM DTT) were obtained by mixing with an equal volume of precipitant consisting of 12 to 14% PEG 6000, 10% Tacsimate, pH 5, at 21°C.

### Structure determination.

Diffraction data were collected on the MX beamlines at the Australian Synchrotron (Clayton, Australia), at 100 K using Blue-Ice (34) and processed with XDS (35). For PFO^N197W^ structure determination, molecular replacement was performed using MOLREP (36) with 1PFO (22, 23). Diffraction data were acquired from DLY crystals containing selenomethionine residues. Experimental phases were determined using the MAD method with AutoRickshaw (37, 38). The data and refinement statistics are given in Table S1 in the supplemental material.

### MD simulations.

Models derived from the PFO^N197high^ structure and the PFO structure (1PFO) were prepared. Each model was solvated and ionized to be electrically neutral with 0.15 M NaCl. Periodic boundary conditions were employed to restrict the simulation to 132 Å by 64 Å by 72 Å with a total of 16,670 TIP3P (transferable intermolecular potential with 3 points) water molecules and 50 Na^+^ and 47 Cl^−^ ions. The total number of atoms in the simulation was 57,367 for PFO and 57,377 for PFO^N197W^. Initial equilibration and simulation were carried out using NAMD (39) on BlueGene/Q architecture for 30-ns simulation with an NPT ensemble (constant number of particles, constant pressure, and constant temperature) at 310 K maintained with a Langevin thermostat. Long-range coulomb forces were computed with the Particle Mesh Ewald method with a grid spacing of 1 Å. Two-femtosecond time steps were used with nonbonded interactions calculated every 2 fs and full electrostatics every 4 fs while hydrogens were constrained with the SHAKE algorithm. The cutoff distance was 12 Å with a switching distance of 10 Å and a pair-list distance of 14 Å. Pressure was controlled to 1 atmosphere using the Nosé-Hoover-Langevin piston method employing a piston 316 period of 100 fs and a piston decay of 50 fs. Trajectory frames were captured every 100 picoseconds. MD trajectories were analyzed and water maps were calculated using VMD (24).

### Accession numbers.

The structures have been deposited in the Protein Data Bank (http://www.rcsb.org/pdb/) under the accession numbers 5DHL and 5DIM for PFO^N197Whigh^ and PFO^N197Wlow^, respectively, and 6NAL for DLY.
