# Supplementary material for: The Structural Basis for a Transition State That Regulates Pore Formation in a Bacterial Toxin
Source: mBio. 2019 Apr 23;10(2):e00538-19. doi: 10.1128/mBio.00538-19 (PMC6479001; doi:10.1128/mBio.00538-19)

**Figure S1.** **PFO and PFO^N197W^ oligomer and pore formation sensitivity to elevated temperature**. PFO or PFO^N197W^ were incubated in solution at the indicated temperatures for 20 minutes and then half of the sample was mixed with cholesterol-rich liposomes and incubated for an additional 30 min at 37°C to allow formation of the oligomeric pore. The remaining sample was used to determine the specific activity of pore formation and the %Act reflects the relative pore-forming activity of the samples at 37°C and 45°C compared to the specific activity of pore formation at 23°C. The oligomer formation in the absence and presence of cholesterol-rich liposomes was evaluated by SDS-AGE. M: monomeric PFO, O: oligomeric PFO. The first lane of each experiment is soluble monomer.


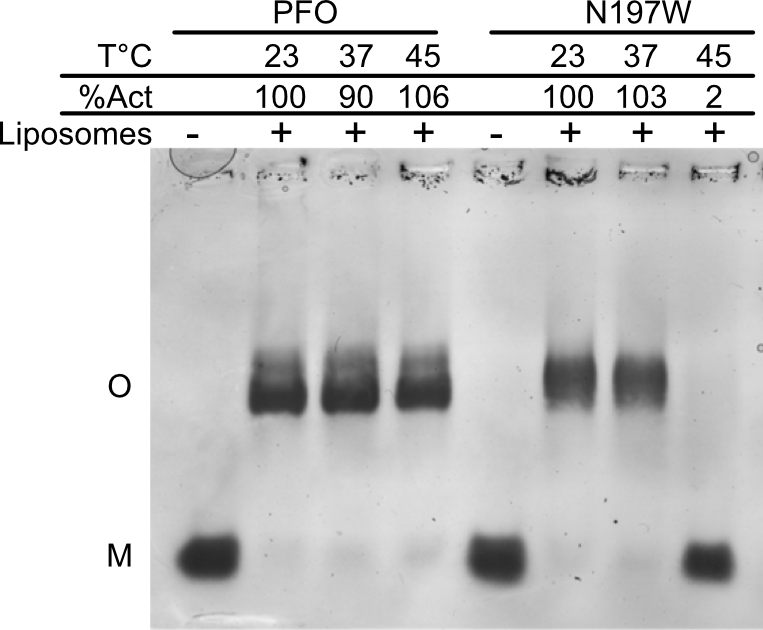

Supplement: FIG S1 [file mBio.00538-19-sf001.docx]
