# Supplementary material for: The Structural Basis for a Transition State That Regulates Pore Formation in a Bacterial Toxin
Source: mBio. 2019 Apr 23;10(2):e00538-19. doi: 10.1128/mBio.00538-19 (PMC6479001; doi:10.1128/mBio.00538-19)
Supplement: TABLE S1 [file mBio.00538-19-st001.docx]

**Table S1. Crystallographic data and refinement statistics**. **Crystal structures of PFO^N197W^ and DLY^.^**

| Crystal form | PFO^N197W high^ | PFO^N197W low^ |  | DLY |  | |  |  |
| --- | --- | --- | --- | --- | --- | --- | --- | --- |
| **Data collection** |  |  |  |  |  | |  |  |
| Space Group | *C2* | *C222_1_* |  | *C2* |  | |  |  |
| Cell dimensions |  |  |  | | |  | |  |
| *a,b,c* (Å) | 161.6, 212.4, 47.0 | 47.0, 207.3, 212.4 | 141.1, 85.4, 102.4 | | |  | |  |
| *β* (**°**) | 97.2 | 90 | 101.1 | | |  | |  |
| Resolution (Å)^1^ | 45.25 – 2.67 | 47.27 – 3.32 | 43.44 – 2.30 | | |  | |  |
| Completeness (%) | 98.7 (94.7) | 100.0 (100.0) | 100.0 (99.9) | | |  | |  |
| I/σ_I_ | 7.9 (1.5) | 9.6 (3.2) | 13.1 (1.2) | | |  | |  |
|  |  |  |  | | |  | |  |
| *R _p. i. m._ (%)* | 7.0 (54.0) | 8.2 (28.5) | 4.6 (71.0) | | |  | |  |
| *CC (1/2)* | 0.995 (0.645) | 0.993 (0.886) | 0.992 (0.572) | | |  | |  |
| Redundancy | 3.8 (3.8) | 7.3 (7.4) | 39.3 (7.6) | | |  | |  |
| Wilson B factor (Å^2^) | 68.8 | 52.3 | 41.8 | | |  | |  |
|  |  |  |  | | |  | |  |
| **Refinement** |  |  |  | | |  | |  |
| Resolution (Å) | 2.67 | 3.32 | 2.30 | | |  | |  |
| No. of reflections | 43968 | 15908 | 53161 | | |  | |  |
| *R*_work_ (%)^3^ / *R*_free_ (%)^2^ | 19.0 / 23.80 | 23.5 / 29.8 | 18.8 / 23.9 | | |  | |  |
| *Non-hydrogen atoms* |  |  |  | | |  | |  |
| Protein | 7376 | 3667 | 7436 | | |  | |  |
| Water | 101 | 3 | 202 | | |  | |  |
| Ligands | 30 | 15 | 23 | | |  | |  |
| Overall B factor (Å^2^) | 59.2 | 64.9 | 53.9 | | |  | |  |
| r.m.s.d. values from ideal |  |  |  | | |  | |  |
| Bond lengths (Å) | 0.015 | 0.010 | 0.015 | | |  | |  |
| Bond angles (°) | 1.16 | 1.47 | 1.43 | | |  | |  |
|  |  |  |  | | |  | |  |
| **Ramachandran plot (%)** |  |  |  | | |  | |  |
| Most favored | 94.8 | 93.0 | 94.7 | | |  | |  |
| Allowed region | 5.2 | 7.0 | 4.7 | | |  | |  |
| Outliers | 0 | 0 | 0.6 | | |  | |  |

^1^Values in parentheses represent the highest resolution shell. ^2^*R*_free_ is based on 5% of the total reflections excluded from refinement. The values in parentheses are for the highest resolution bin (approximately 0.1 Å width).

1. Laskowski RA, Swindells MB. 2011. LigPlot+: multiple ligand-protein interaction diagrams for drug discovery. J Chem Inf Model 51:2778-86.
