# Supplementary material for: The Structural Basis for a Transition State That Regulates Pore Formation in a Bacterial Toxin
Source: mBio. 2019 Apr 23;10(2):e00538-19. doi: 10.1128/mBio.00538-19 (PMC6479001; doi:10.1128/mBio.00538-19)
Supplement: FIG S2 [file mBio.00538-19-sf002.docx]

**Figure S2.** **The environment of N197 and N197W**. LIGPLOT(1) representations of the environment around residue 197 in wild-type PFO (PDB id: 1PFO) and in PFO^N197Whigh^ (2.7Å resolution, monomer B). Hydrogen bonds are indicated by green dashed lines and radiating red lines are hydrophobic interactions.
