# Supplementary material for: The Structural Basis for a Transition State That Regulates Pore Formation in a Bacterial Toxin
Source: mBio. 2019 Apr 23;10(2):e00538-19. doi: 10.1128/mBio.00538-19 (PMC6479001; doi:10.1128/mBio.00538-19)

**Figure S3. MD simulations showing the impact of the PFO^N197W^ mutation on the D3-D1,2 interface waters***.* In (**A**) the overlaid models of PFO wildtype (pink) and PFO^N197W^ (green) are shown with the associated water density maps derived from the MD simulation near N197; solid cyan represents the PFO water map and transparent yellow shows the PFO^N197W^ water map. The PFO water map contains a region of high water density adjacent to the N197 side-chain, which is not present in PFO^N197W^ mutant (red box). In (**B**) a top-down view of the PFO wild-type and associated water map, cut-away at a plane just above the N197 side-chain shows the two regions of highly stable water density that form a solvent chain from the side-chain of N197 to pin the backbone of residues S93 and L114. Two of the crystallographic waters that sit along this solvent chain (683 and 637 in 1PFO) are shown. This stable water chain is absent from the PFO^N197W^ simulation due to the loss of the interaction with the N197 side-chain that exists in the wild-type protein.


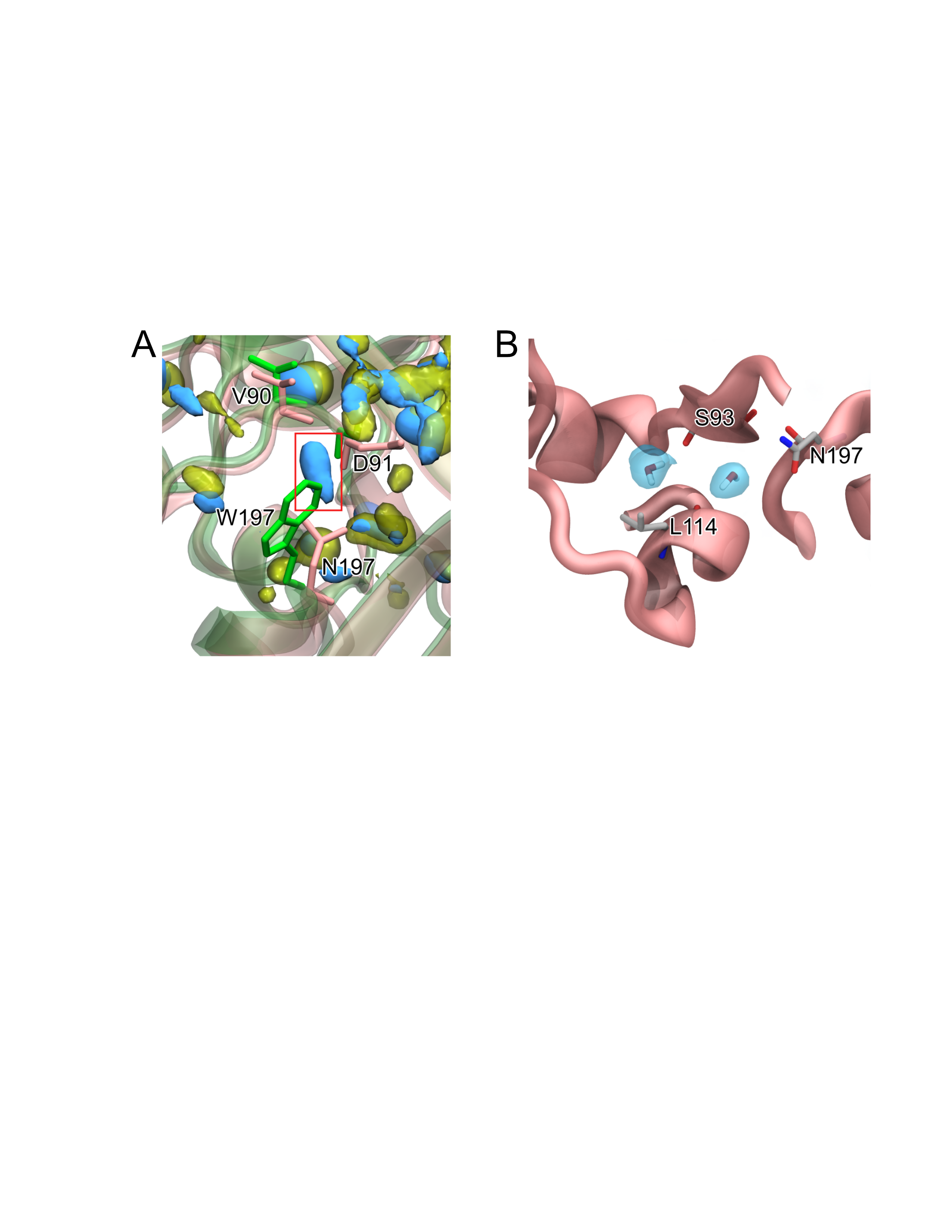

Supplement: FIG S3 [file mBio.00538-19-sf003.docx]
